# Perampanel, Brivaracetam, Cenobamate, Stiripentol, and Ganaxolone in Lennox-Gastaut Syndrome: A Comprehensive Narrative Review

**DOI:** 10.3390/jcm14176302

**Published:** 2025-09-06

**Authors:** Debopam Samanta

**Affiliations:** Division of Child Neurology, Department of Pediatrics, University of Arkansas for Medical Sciences, 1 Children’s Way, Little Rock, AR 72202, USA; dsamanta@uams.edu

**Keywords:** developmental and epileptic encephalopathy, epilepsy, seizure, AED, medicines

## Abstract

**Background:** Lennox–Gastaut syndrome (LGS) is a severe childhood-onset developmental and epileptic encephalopathy characterized by treatment-resistant seizures and significant morbidity. Despite multiple approved anti-seizure medications (ASMs), optimal seizure control remains elusive. This has led to ongoing interest in newer ASMs, including those not specifically approved for LGS. This review evaluates the emerging evidence on the use of these agents in LGS management. **Methods:** We conducted a comprehensive literature search of PubMed, Web of Science, and Embase to identify studies examining perampanel, brivaracetam, cenobamate, ganaxolone, and stiripentol in LGS populations. Both randomized controlled trials and observational studies were included. **Results:** Perampanel was studied in approximately 300 patients across one Phase 3 trial and seven observational studies, showing responder rates of 26–69% with particular efficacy for generalized tonic–clonic and myoclonic seizures, though behavioral side effects (irritability, aggression) were dose-related concerns. Brivaracetam demonstrated inconsistent efficacy in 59 patients across six studies (0–61.5% responder rates) but offered better behavioral tolerability than levetiracetam. Cenobamate showed exceptional promise in 223 patients across seven studies with 50–85% responder rates and significant polypharmacy reduction, though requiring careful titration. Ganaxolone demonstrated efficacy in LGS-like CDKL5 deficiency phenotypes with 28.2% drop seizure reduction versus placebo. Stiripentol showed potential benefit for generalized seizures in limited LGS data. **Conclusions:** Several newer ASMs show therapeutic promise in LGS. Perampanel offers the most extensive evidence base, cenobamate demonstrates exceptional efficacy potential, while brivaracetam provides an alternative for levetiracetam-intolerant patients. Further controlled studies are needed to define optimal treatment algorithms.

## 1. Introduction

Lennox–Gastaut syndrome (LGS) is one of the most challenging developmental and epileptic encephalopathies (DEEs) of childhood, accounting for approximately 1–2% of all epilepsy cases and 3–4% of pediatric epilepsy populations [1]. It is characterized by multiple, often treatment-resistant seizure types—most notably tonic seizures—alongside significant cognitive and behavioral impairments [2]. According to the recent International League Against Epilepsy (ILAE) definition, a hallmark of LGS is its characteristic interictal EEG pattern, marked by slow spike–wave complexes and generalized paroxysmal fast activity [2].

Beyond the classic triad of polymorphous seizures, cognitive impairment, and characteristic EEG findings, individuals with LGS experience substantial disease burden [3,4,5,6]. More than 50% will have at least one episode of status epilepticus, and many face significantly impaired health-related quality of life and markedly elevated rates of premature mortality [6]. Treatment resistance—across anti-seizure medications (ASMs), dietary therapies, neuromodulation, and surgical interventions—further exacerbates this burden, leading to persistent functional disability, psychological distress for families, and disproportionately high healthcare utilization [7,8,9,10,11,12,13]. Currently, eight ASMs hold regulatory approval specifically for LGS. Valproate, although not formally approved for this indication, remains a first-line treatment due to its broad-spectrum efficacy [8]. Nevertheless, despite this array of approved therapies and the off-label use of more than 20 additional ASMs, seizure control remains out of reach for most patients [9,14,15]. (Table 1) This enduring therapeutic gap has driven ongoing interest in evaluating newer ASMs for their potential role in managing LGS [16].

**Figure 1:**
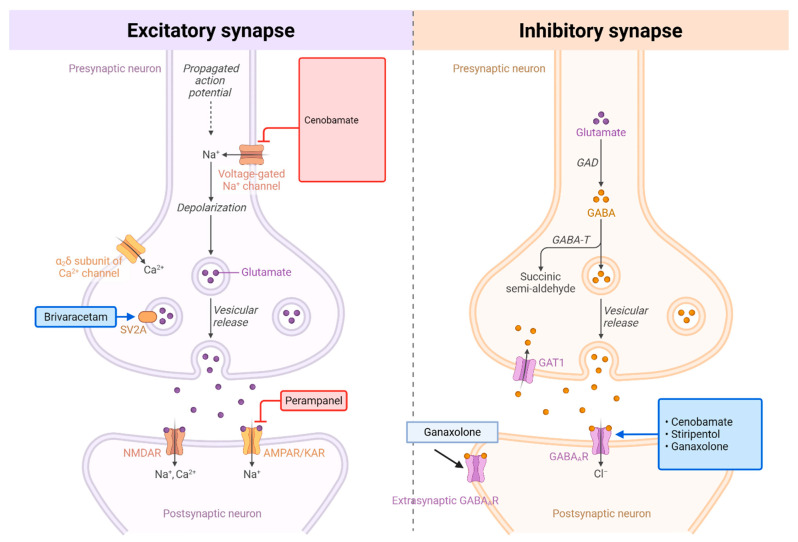
Mechanisms of Action: Perampanel, Brivaracetam, Cenobamate, Stiripentol, and Ganaxolone. Comparison of excitatory and inhibitory synaptic transmission and their pharmacological targets. (Left) Excitatory synapse: Upon arrival of an action potential, depolarization opens voltage-gated Na^+^ channels, leading to further depolarization and activation of voltage-gated Ca^2+^ channels (α_6_δ subunit shown). Calcium influx triggers vesicular release of glutamate neurotransmitter. Glutamate binds to postsynaptic ionotropic receptors (NMDAR and AMPAR/KAR), allowing Na^+^ and Ca^2+^ influx that depolarizes the postsynaptic neuron. Key pharmacological agents include: cenobamate (voltage-gated Na^+^ channel blocker), brivaracetam (SV2A modulator affecting vesicular release), and perampanel (AMPAR antagonist). (Right) Inhibitory synapse: Similarly triggered by action potential propagation, this synapse releases GABA neurotransmitter through vesicular exocytosis. GABA is synthesized from glutamate by glutamic acid decarboxylase (GAD). Released GABA binds to postsynaptic GABA_A_ receptors, opening Cl^−^ channels that hyperpolarize and inhibit the postsynaptic neuron. GABA is metabolized by GABA transaminase (GABA-T) to succinic semi-aldehyde. Synaptic GABA levels are regulated by reuptake through GAT1 transporters and extrasynaptic GABA_A_ receptors provide tonic inhibition. Pharmacological modulators include ganaxolone (positive allosteric modulator of GABA_A_ receptors) and agents that target cenobamate, stiripentol, and ganaxolone pathways. Both synaptic types represent critical targets for antiepileptic drugs that modulate the excitation-inhibition balance in neural circuits. Created with BioRender.com.

Over the past 15 years, ten ASMs have gained regulatory approval [17]. Among these, cannabidiol and fenfluramine have received specific indications for LGS [8,18,19]. Others—such as ezogabine (withdrawn due to safety concerns including retinal toxicity), eslicarbazepine (relatively contraindicated due to its sodium channel blockade worsening absence and myoclonic seizures in LGS), and everolimus (primarily indicated for tuberous sclerosis complex-related epilepsy)—have more limited relevance in LGS [17]. (Figure 1) In contrast, several recently introduced ASMs—perampanel, brivaracetam, stiripentol, cenobamate, and ganaxolone—have shown promise but have yet to be systematically evaluated in this context [17]. Although the precise mechanisms by which these five ASMs act remain incompletely understood, as with epilepsy in general, LGS is fundamentally characterized by an imbalance between neuronal excitation and inhibition. Some ASMs primarily reduce excitatory neurotransmission: brivaracetam binds selectively to synaptic vesicle protein 2A (SV2A), modulating presynaptic neurotransmitter release and thereby diminishing glutamate-mediated excitation, whereas perampanel acts postsynaptically as an AMPA receptor antagonist, blocking glutamate-driven depolarization. Others predominantly enhance inhibitory signaling through GABAergic mechanisms: stiripentol increases inhibitory tone via positive allosteric modulation of GABA_A_ receptors and inhibition of GABA reuptake, while ganaxolone, a synthetic neurosteroid, positively modulates GABA_A_ receptors at both synaptic and extrasynaptic sites, producing sustained inhibitory effects. Cenobamate uniquely engages both pathways, reducing excitation by preferentially inhibiting persistent sodium currents while simultaneously enhancing inhibition through positive allosteric modulation of GABA_A__ receptors.

Despite growing clinical interest, no prior review has synthesized the available data on these five newer ASMs specifically for LGS. Addressing this gap, the present review offers a comprehensive appraisal of their efficacy, seizure type-specific responses, safety profiles, and potential roles in LGS treatment strategies.

## 2. Methods

A comprehensive literature search was conducted using PubMed, Web of Science, and Embase databases to identify relevant studies examining the use of perampanel, brivaracetam, cenobamate, ganaxolone, and stiripentol in individuals with LGS. Eligible studies included those that specifically reported clinical outcomes in patients diagnosed with LGS or with phenotypes consistent with LGS. (Table 2) Both randomized controlled trials (RCT) and observational studies were included to capture the full range of available evidence. (Figure S1) For each ASM, the following parameters were systematically analyzed: Efficacy outcomes, including responder rates (≥50% seizure reduction), seizure freedom rates, and seizure type-specific responses, and safety and tolerability, including frequency and type of reported adverse events.

The studies included in this review are largely retrospective observational analyses, with a few exceptions: one RCT (prematurely terminated) and one prospective study of perampanel, one prospective study of stiripentol (available only in abstract form), and a post hoc RCT of ganaxolone (also available in abstract form). All cenobamate studies were retrospective, including one national claims-based analysis. Study settings varied: perampanel reports originated from China (n = 1), France (n = 2), Italy (n = 1), Spain (n = 2), and multicountry cohorts (n = 2), with four single-center and four multicenter designs. Brivaracetam studies came from Germany (n = 1), Spain (n = 2), Argentina (n = 1), Israel (n = 1), and one multicountry cohort, comprising two single-center and four multicenter analyses. Cenobamate was examined in studies from the United States (n = 3), Italy (n = 1), Spain (n = 1), and Germany (n = 1), with three single-center and three multicenter cohorts (including the claims-based study). Stiripentol studies were conducted in France (n = 2) and Spain (n = 1), with two single-center and one multicenter design. Ganaxolone was evaluated in a multicenter trial. Further clinical details such as participant level clinical details, seizure duration, hospitalization, status epilepticus frequency, or concurrent maintenance/rescue therapy were not consistently reported in the LGS subgroups, limiting deeper contextual characterization.

Table 3 provides a comparative overview of the mechanisms, dosing, formulations, pharmacokinetics, interactions, indications, side effects, age approvals, scheduling, and dosing considerations of Perampanel, Brivaracetam, Cenobamate, Stiripentol, and Ganaxolone. In separate sections, this review will discuss the efficacy, safety, and clinical role of these ASMs in the treatment of LGS.

## 3. Perampanel

Perampanel is a selective, non-competitive α-amino-3-hydroxy-5-methyl-4-isoxazolepropionic acid (AMPA) receptor antagonist. Perampanel is approved as adjunctive therapy for focal and primary generalized tonic–clonic seizures (GTCS) in patients aged 12 and older, and as monotherapy for focal seizures in patients aged 4 and older.

### 3.1. Efficacy in LGS

Perampanel has been studied in LGS through one pivotal Phase 3 RCT and seven observational studies (three single-center and four multicenter), collectively involving approximately 300 patients—the most extensive dataset among the ASMs reviewed [20]. The RCT was terminated early due to recruitment challenges and disruptions from the COVID-19 pandemic, which limited its statistical power and generalizability [20]. Although the trial did not meet its primary endpoint of significant reduction (23.1% vs. 4.5%, *p* = 0.107) in protocol-defined drop seizures (atonic, tonic, and myoclonic seizures that led to or could have led to a fall), perampanel achieved statistically significant reductions in broader seizure categories [20]. Specifically, atonic/tonic/tonic–clonic seizures decreased by 48.6% with perampanel compared to −1.7% with placebo (*p* = 0.001), and all countable motor seizures declined by 44.0% versus −0.6% (*p* = 0.017) [20]. The 50% responder rate for all motor seizures was also significantly greater with perampanel (45.5% vs. 20.6%, *p* = 0.0167), with efficacy sustained for up to 71 weeks [20].

Observational studies highlight the variable efficacy of perampanel in LGS, with 50% responder rates ranging from 32.4% to 69.2% [21,22,23,24,25]. Only one was prospective, reporting a 69.2% responder rate in 13 children over a median follow-up of 10.8 months [21]. A large retrospective study of 71 adults with LGS reported a 65% responder rate, with 35.2% achieving ≥75% seizure reduction [23]. Several studies involved mixed epilepsy populations with LGS subgroups, reporting responder rates between 40% and 66% over 12 months [22,25]. In a multicenter retrospective study of 87 LGS patients, 41.4% responded over a median follow-up of 11 months; however, 36.1% of initial responders experienced seizure relapse—defined as a new seizure in a previously seizure-free patient or a ≥50% increase in seizure frequency—after a median of 21 months, reducing the sustained responder rate to 26.4% [24].

Although seizure freedom was <4% in the RCT, two observational studies reported seizure freedom or near-freedom (≥90% reduction) rates of 17–33% over 12–14 months of follow-up [23,25].

Seizure-type–specific responses to perampanel were occasionally reported, including in the aforementioned RCT and several observational studies. In one study of 64 patients (31 with “pure” LGS and 33 LGS-like), seizure freedom at 12 months was achieved in 35% of those with GTCS (*p* < 0.001), 17% with tonic seizures (*p* = 0.016), and 37% with seizure clusters (*p* < 0.001) [26]. A significant shift from daily to less frequent seizures was observed in 22% of patients with tonic seizures and 19% with focal seizures (*p* = 0.004 and *p* = 0.02, respectively) [26]. Seizure freedom was also reported for other types—7% for focal seizures, 28% for atypical absence, 6% for tonic seizures, and 18% for myoclonic seizures—though these did not reach statistical significance [26]. In another study, 41.4% of patients responded for all countable motor seizures, with 61% of these also showing a response for drop seizures [24]. Additional studies suggest that perampanel may have sustained and greater efficacy for GTCS and myoclonic seizures. Yamagishi et al. reported seizure-type–specific response rates at 36 and 60 months, respectively: atypical absence (27.3%, 30.0%), tonic (33.3%, 44.4%), focal (20.0%, 33.3%), GTCS (66.7%, 80.0%), myoclonic (57.1%, 66.7%), and atonic (37.5%, 28.6%). These findings highlight the durability of perampanel’s effects, particularly for GTCS and myoclonic seizures [27].

Cognitive and behavioral outcomes are infrequently reported in perampanel studies. In one study, where 69.2% of 13 LGS patients were responders, 53.8% were informally reported to have improved cognition and behavior [21]. In another study of 71 adults, four patients described positive changes in behavioral and psychological well-being [23]. These limited findings suggest potential non-seizure benefits, although robust data are lacking.

### 3.2. Safety and Tolerability

Retention rates varied across studies but generally ranged from 60 to 70% over a one-year period. In individual studies, retention was reported as 62% over 14 months with 24% discontinuing due to adverse events (AE), 69.8% over 11 months with no discontinuations due to AE, and 66.7% over 12 months with 21% due to AE [21,23,26]. In the RCT, 5 of 34 participants (14.7%) discontinued during the 18-week core study [20].

Safety data from the Phase 3 trial revealed that 85.3% of perampanel-treated patients experienced one or more treatment-emergent adverse events (TEAEs), compared to 72.2% in the placebo group [20]. The most common AEs were somnolence (23.5%), irritability (14.7%), upper respiratory infections (11.8%), and decreased appetite (11.8%). Psychiatric TEAEs occurred in 35.3% of perampanel patients, most commonly irritability (14.7%) and agitation (5.6%) [20].

Behavioral AEs have been reported in 8.5–32% of patients in observational studies and are more frequent at doses ≥6 mg/day [23]. For instance, AEs occurred in 52% of patients, including behavioral issues in 32%, seizure aggravation in 8.5%, and somnolence in 5.6% [23]. In another study, 6 of 13 patients (46.2%) experienced AEs—mainly behavioral changes and fatigue—all manageable by reducing the dose from 8 mg to 6 mg [21]. In a retrospective multicenter study, AEs were observed in 44.8% of patients, with behavioral disturbances (n = 19) and somnolence (n = 11) being most common [24]. These AEs were associated with shorter epilepsy duration, a high number of prior ASMs, and rapid titration [24]. Of the 39 patients who experienced AEs, 30 discontinued perampanel, while the remainder improved with dose reduction [24]. Another study reported AEs in 52.9% of patients, including mood changes (21.8%) and somnolence (17.2%) [26]. Overall, 29 patients discontinued perampanel: 18 (21%) due to AEs, 8 (9%) due to lack of efficacy, and 3 (3%) due to seizure worsening.

### 3.3. Current Clinical Role

Perampanel holds promise in the treatment of LGS, offering several advantages: a novel mechanism of action as an AMPA receptor antagonist, once-daily dosing supported by a long elimination half-life (~105 h), and favorable efficacy for GTCS, myoclonic, and atypical absence seizures [28,29]. The long half-life not only minimizes the impact of missed doses but also eliminates the need for tapering upon discontinuation.

Although perampanel has been studied in an RCT specifically for LGS, the trial failed to meet its primary endpoint using the prespecified drop seizure definition [20]. A post hoc reclassification of definition of drop seizures to achieve statistical significance illustrates a common challenge in LGS research: seizure heterogeneity often necessitates flexible outcome definitions, but such flexibility can introduce bias [16,20,30]. Observational studies have shown variable results—likely reflecting differences in study design, patient populations, co-administered medications, and outcome measures—but generally report seizure responder rates of approximately 40–50% [16].

Additional advantages of perampanel include its availability as an oral suspension, which facilitates use in pediatric populations. Beyond chronic seizure management, perampanel may have potential utility in treating refractory or super-refractory status epilepticus, a common complication in LGS [31,32]. However, the lack of an intravenous formulation currently limits its use in acute settings, although development is ongoing [33]. Perampanel also demonstrates an early onset of action. Reductions in drop seizure frequency have been observed as early as 3–4 weeks, consistent with its pharmacokinetics, as steady-state levels are typically achieved within three weeks of once-daily dosing [20].

Some caution is warranted during its use. The psychiatric side effect profile of perampanel necessitates careful patient selection and monitoring, particularly in individuals with pre-existing behavioral concerns. In clinical trials for partial-onset seizures, psychiatric AEs were reported in 17% of patients on 8 mg/day and 22% on 12 mg/day, compared to 12% in the placebo group [34]. These events were dose-related and most commonly emerged within the first six weeks of treatment, though they could occur up to 37 weeks after initiation. Our review also found a notable risk of behavioral side effects in patients with LGS, who often have baseline behavioral comorbidities.

Additionally, perampanel has important drug–drug interactions. It may reduce the effectiveness of contraceptives containing levonorgestrel and is subject to pharmacokinetic interactions with CYP3A4 inducers (e.g., carbamazepine, phenytoin, oxcarbazepine), which can lower perampanel plasma levels and potentially reduce efficacy [35].

## 4. Brivaracetam

Brivaracetam is a third-generation racetam derivative and a 4-n-propyl analog of levetiracetam [36]. Although structurally related, brivaracetam exhibits approximately 20-fold higher affinity for synaptic vesicle protein 2A (SV2A), offering greater selectivity and enhanced brain permeability compared to levetiracetam [36]. It is approved for both monotherapy and adjunctive therapy in the treatment of focal seizures in patients aged 1 month and older.

### 4.1. Efficacy in LGS

The evidence supporting brivaracetam’s use in LGS is limited and primarily derived from retrospective observational studies, with no available RCTs or prospective studies. Among six published studies (four multicenter and two single-center), a total of 59 patients with LGS were evaluated, with reported 50% responder rates ranging from 0% to 61.5%.

Several studies showed limited or no efficacy. In a study involving 93 pediatric epilepsy patients (including 8 with LGS), the overall 12-month responder rate was 17.2%, but none of the LGS patients responded [37]. Similarly, zero responders were reported among six LGS patients in a Spanish multicenter study [38]. Another study in children reported only one responder among five LGS patients (20%) over 12 months [39]. Better results were reported in a cohort of 31 patients, with two of five LGS patients (40%) achieving greater than 50% seizure reduction [40]. Another study in 44 DEEs (20 with LGS) tracked responders longitudinally and showed declining efficacy over time: responder rates were 36% at 3 months, 43% at 6 months, and 20% at 12 months [41]. The most promising findings came from a multicenter study including 42 pediatric patients with DEEs, of whom 13 had LGS. Among these, 8 (61.5%) were responders, and one achieved seizure freedom. EEG improvement greater than 50% was observed in four patients, including one with 80% improvement [42].

Seizure-type–specific analysis revealed differential efficacy. The highest response rates were seen in focal seizures (83.6%), followed by myoclonic seizures (76%), atonic seizures (73.9%), tonic seizures (69%), GTCS (66.6%), epileptic spasms (61.5%), and atypical absence seizures (47.8%) [42]. Additionally, one LGS patient experienced worsening of tonic seizures four months after initiating brivaracetam. However, another study found that seizure types with generalized semiology—such as GTCS, myoclonic, and absence seizures—tended to show better responses, with 50% responder rates exceeding 50% [41]. Additionally, 57% of patients with drop seizures achieved greater than 50% seizure reduction in another study [40].

### 4.2. Safety and Tolerability

Brivaracetam generally demonstrates a favorable safety profile, especially in comparison to levetiracetam. In one study, mild AEs such as drowsiness, irritability, and decreased appetite were reported in 16.6% of patients, none of whom discontinued treatment [42]. Importantly, unlike levetiracetam, brivaracetam does not inhibit AMPA receptors, which may contribute to its improved behavioral tolerability [41].

Nonetheless, psychiatric and behavioral AEs can still occur, particularly in individuals with intellectual disability. In a multicenter study, TEAEs occurred in 16% of patients, and 9% discontinued due to AEs [41]. Notably, psychobehavioral TEAEs were present in 14% of patients on brivaracetam, compared to 32% of patients previously exposed to levetiracetam, supporting its utility as a substitute in individuals with behavioral complications [41]. Furthermore, an overnight switch from levetiracetam to brivaracetam (in 29 patients, 30.1%) resulted in behavioral improvement in 5 patients (17.2%) [37]. However, in a separate cohort, 13 of 33 individuals with intellectual disability developed new behavioral issues within six months of brivaracetam initiation, especially when doses approached 100–200 mg/day, suggesting that upward titration should be approached with caution [43].

A decline in retention rates to 40–50% over one year has been noted in several studies. In one study, retention dropped from 80% at 3 months to 45% at 12 months [37]. Similarly, Willems et al. reported retention rates of 65%, 52%, and 41% at 3, 6, and 12 months, respectively [41]. Another study found that approximately half of the patients with DEEs continued brivaracetam during a 12-month follow-up period [38].

### 4.3. Current Clinical Role

Brivaracetam offers several potential advantages for managing LGS, particularly in patients who are unable to tolerate levetiracetam due to behavioral AEs. Its favorable pharmacokinetic profile, availability in an IV formulation, and the option to initiate treatment at full therapeutic doses make it especially appealing in acute or severe seizure settings. In clinical practice, direct substitution from levetiracetam to brivaracetam at a dose ratio of 10:1 to 15:1 is generally feasible [44].

Interestingly, prior failure of levetiracetam does not necessarily predict lack of response to brivaracetam in LGS, at least based on limited data. In the study by Ferragut et al., 27.9% of patients who did not respond to levetiracetam showed a favorable response to brivaracetam [39]. The availability of an IV formulation has also prompted investigation into its use for status epilepticus, though evidence in this setting remains limited [45].

However, brivaracetam is a controlled substance and exhibits more drug–drug interactions than levetiracetam [46]. Its clearance may be increased by enzyme inducers, potentially requiring dose adjustments [46]. While overall efficacy in LGS appears modest—with 4 out of 6 studies reporting responder rates of only 0–20%—certain patients, particularly those with myoclonic or atonic seizures, may still experience meaningful benefit.

## 5. Cenobamate

Cenobamate is an alkyl-carbamate ASM with a dual mechanism of action: preferential inhibition of persistent sodium currents and positive allosteric modulation of GABA receptors. Approved by the FDA in November 2019 for treatment-resistant focal epilepsy in adults, cenobamate has demonstrated unparalleled efficacy in clinical trials, achieving seizure freedom rates between 10 and 20%—higher than any other ASM introduced in the past three decades [47,48,49,50].

### 5.1. Efficacy in LGS

Although evidence for cenobamate in LGS remains limited, emerging data from several small studies and real-world reports (223 patients across seven studies) suggest impressive efficacy in this challenging population. A study of 36 pediatric and adult patients with LGS (median age 15.5 years) reported outcomes after a median of 23 months of cenobamate treatment. [51]. Of these, 86% achieved >50% seizure reduction, including ≥75% reduction in 61% and seizure freedom in 14% [51]. These outcomes substantially exceed typical response rates seen with other ASMs in LGS. Moreover, 75% of participants were able to reduce concomitant medications—most notably cannabidiol (n = 19) and clobazam (n = 21) [51].

Supporting these findings, a multicenter Spanish study reported a 46.2% responder rate (≥50% seizure reduction) among 18 patients (mean age 25.2 years) treated with cenobamate over 12 months [52]. Notably, 12.5% of patients became seizure-free, and 23.1% achieved ≥75% seizure reduction [52]. Polypharmacy was significantly reduced: while 47% of patients were initially on ≥4 co-ASMs, this figure dropped to 11% after 12 months [52]. Concurrently, the proportion of patients on just one or two co-ASMs increased from 18% to 33% [52]. The most commonly withdrawn ASMs included sodium channel blockers (e.g., lamotrigine and carbamazepine) and clobazam, resulting in a 26.4% reduction in total ASM burden [52].

A German multicenter study involving 41 patients with DEEs, including 31 with LGS, reported responder rates of 56.1% at 3 months, 37.1% at 6 months, and 34.5% at 12 months [53]. In this cohort, 8 patients were under 18 years old, and outcomes did not differ significantly between pediatric and adult patients [53]. Impressively, three patients remained seizure-free through 12 months, and all demonstrated a reduction in overall drug load [53].

Further supporting evidence comes from a single-center German study involving 5 adults with LGS, in which 4 patients (80%) achieved ≥50% seizure reduction over a median follow-up of 22 months [54]. Similarly, a case series by Falcicchio et al. described four adult LGS patients with seizure frequency reductions of 25–74% at 12 months; two achieved ≥50% seizure reduction [55]. Another case series of 4 patients with LGS also showed two with >50% seizure reduction [56].

Data from these studies also highlight seizure-type specificity. Cenobamate was especially effective against tonic and GTCS, followed by atonic seizures [52]. In one study, approximately 25% of patients achieved GTCS freedom for up to 12 months [53]. Another study involving four patients reported varied responses: one patient experienced >50% reduction in both focal and tonic seizures, another had a 50% reduction in GTCS, and a third showed a 42.8% reduction in absence seizures [56].

Real-world effectiveness of cenobamate was further supported by a national claims-based study involving 125 children with LGS (mean age 13.6 years, range 2–17) [57]. During treatment, inpatient days decreased from 4.5 to 1.8 per 100 days, and emergency room visits declined from 0.3 to 0.2 per 100 days [57]. Among patients without a history of status epilepticus, new episodes occurred in 20.6% during prior therapies compared to only 7.7% during cenobamate treatment [57].

Changes in global functioning were infrequently reported. In one study using the Clinical Global Impression of Change (CGI-C), 12.2% of patients were rated as “very much improved,” 22% as “much improved,” and 12.2% as “minimally improved,” while 2.24% were rated “minimally worse” and 2.4% “much worse” [53].

### 5.2. Safety and Tolerability

In terms of safety, cenobamate’s AE profile is consistent with findings from its initial approval trials. In one study, AEs were reported in approximately two-thirds of patients, with somnolence being the most common (44%), followed by other central nervous system symptoms [51]. Two patients experienced seizure worsening. In the small German series (n = 5), all participants reported side effects, including gait disturbance (n = 3) and fatigue (n = 2) [54]. All 4 patients showed sedation or ataxia in another study [56]. In the larger German cohort (n = 41), 58.5% experienced AEs—primarily CNS-related (46.3%)—leading to drug withdrawal in 17.1% of cases. Psychobehavioral TEAEs were infrequent (12.2%) [53].

The most serious safety concern associated with cenobamate is the risk of drug reaction with eosinophilia and systemic symptoms (DRESS). Although this risk was initially observed during early clinical development, it has been largely mitigated by implementing a slow titration protocol. The currently recommended titration schedule begins at 12.5 mg daily for two weeks, with increases of 12.5–25 mg every two weeks until the target dose is achieved. Among the studies reviewed, only one case (out of 41 patients) of rash and pruritus requiring discontinuation by day 43 was reported in the German cohort [53].

### 5.3. Current Clinical Role

Cenobamate is among the most promising newer ASMs for the treatment of LGS, owing to its potent efficacy, notable rates of seizure freedom, and potential to reduce polypharmacy—an especially valuable benefit in a population often burdened by complex medication regimens. Its dual mechanism of action—modulating persistent sodium channels and enhancing GABAergic inhibition—aligns well with the pathophysiology of LGS, where both excitatory and inhibitory imbalances contribute to seizure generation. Cenobamate may offer particular advantages in certain genetic subtypes of LGS, such as gain-of-function variants in *SCN8A*. In one study, 8 out of 12 patients achieved >50% seizure reduction over 17 months of treatment, with 80% also showing improvements in non-seizure-related outcomes [58].

Pharmacokinetically, cenobamate has a long half-life of 50–60 h, supporting convenient once-daily dosing [59].

Despite these advantages, several limitations must be considered. Cenobamate is currently approved only for adult use, limiting access for the predominantly pediatric LGS population. Its use requires careful titration and attention to drug–drug interactions. It inhibits CYP2C19—raising serum levels of phenytoin, phenobarbital, and the active metabolite of clobazam—and induces CYP3A4, potentially reducing the efficacy of oral contraceptives and lowering lamotrigine concentrations [59]. As a Schedule V controlled substance, it also carries additional regulatory constraints.

Nonetheless, for adolescent and adult patients with refractory LGS—particularly those with persistent seizures despite multiple therapies—cenobamate should be strongly considered. Its ability to achieve robust seizure control while potentially reducing medication burden represents a meaningful advance in the management of this complex epilepsy syndrome.

## 6. Ganaxolone

Ganaxolone is a neuroactive steroid that acts as a positive allosteric modulator of GABA receptors, binding at a site distinct from benzodiazepines or barbiturates [60]. It is currently approved for the treatment of seizures associated with cyclin-dependent kinase-like 5 (CDKL5) deficiency disorder (CDD) in patients aged 2 years and older. Its potential relevance to LGS stems from the fact that many individuals with CDKL5 deficiency exhibit LGS-like phenotypes.

### 6.1. Efficacy in LGS

The primary evidence supporting ganaxolone’s use in LGS comes from a post hoc analysis of a Phase 3 RCT originally designed to evaluate its efficacy in CDD. Given that CDKL5-related epilepsy often evolves into an LGS-like presentation, this subgroup analysis is clinically relevant. In the Marigold study, which enrolled 101 patients aged 2–19 years with CDD and ≥16 major motor seizures (MMS) per month, ganaxolone treatment led to a median 30.7% reduction in 28-day MMS frequency compared to a 6.9% reduction with placebo (*p* = 0.0036) [61]. The post hoc analysis focused on patients within this trial exhibiting LGS-like features, including 17 patients treated with ganaxolone and 20 with placebo [62]. In this subgroup, the median percent reduction in drop seizure frequency was 28.2% with ganaxolone versus 2.8% with placebo, a median difference of 29.2% [62]. CGI scores also favored ganaxolone: 69.3% of caregivers and 71.4% of clinicians rated patients as minimally improved or better, compared to 47.4% and 36.8% with placebo, respectively [62].

Additional support comes from a small prospective cohort study of three patients with LGS—two with *PCDH19* and one with a *CDKL5* pathogenic variants [63]. Both patients with *PCDH19* pathogenic variant achieved 75–80% seizure reduction, while the patient with *CDKL5* pathogenic variant experienced >50% seizure reduction over 8–12 weeks of treatment [63]. CGI-I and CGI-P evaluations indicated much improvement in global function for all three patients [63].

Long-term data from the open-label extension of the Marigold study further support sustained benefit [64]. At two years, the median reduction in MMS frequency was 48.2% (n = 50), with 46.0% of patients achieving ≥50% reduction and 24.0% reaching ≥75% reduction [64]. Notably, 81.6% of caregivers reported improvement in seizure-related outcomes [64]. But specific data in LGS population are lacking.

### 6.2. Safety and Tolerability

Ganaxolone has demonstrated a favorable safety and tolerability profile. In the original RCT, TEAEs occurred in 86% of ganaxolone-treated patients and 88% of those receiving placebo [61]. The most commonly reported TEAEs more frequent than placebo included somnolence, pyrexia, and upper respiratory tract infections [61]. Serious AEs were reported in 12% of ganaxolone patients versus 10% of placebo recipients [61]. In the long-term extension phase, the most frequent treatment-related AEs were somnolence (17.0%), seizure (11.4%), and decreased appetite (5.7%) [64]. The overall discontinuation rate due to AEs remained relatively low at 11.4% over the extended treatment period [64].

### 6.3. Current Clinical Role

Ganaxolone’s efficacy in LGS-like phenotypes within CDKL5 deficiency disorder offers proof-of-concept for its potential application in classic LGS. The sustained long-term efficacy and high caregiver satisfaction further support its clinical utility. However, its current FDA approval is restricted to CDD, which limits its accessibility for broader LGS populations.

Although ganaxolone was previously granted Orphan Drug designation for LGS, it is unclear whether further trials specifically targeting LGS populations are planned. For now, ganaxolone should be considered in LGS patients with confirmed CDKL5 mutations or those with LGS-like phenotypes secondary to this genetic disorder. Its use in typical LGS patients would remain off-label pending additional clinical evidence.

## 7. Stiripentol

Stiripentol is a unique ASM with multiple mechanisms of action, including direct positive allosteric modulation of GABA receptors, inhibition of GABA reuptake, lactate dehydrogenase inhibition, and blockade of T-type calcium channels [65]. Stiripentol is approved for the treatment of seizures associated with Dravet syndrome in patients aged 6 months and older, in combination with clobazam, based on evidence from multiple RCTs and real-world studies. Although studies in non-Dravet populations are limited, emerging evidence suggests stiripentol may have equivalent efficacy in broader DEE populations, although efficacy in LGS specifically remains underexplored.

### 7.1. Efficacy in LGS

The evidence supporting stiripentol’s use in LGS primarily comes from small clinical studies involving LGS or LGS-like patients within broader DEE cohorts. Soto-Insuga et al. studied 17 non-Dravet patients, including 4 with LGS, and found that after three months of adjunctive stiripentol treatment, 76.5% of patients showed improvement in seizure characteristics (number, duration, and/or intensity) [66]. Seizure frequency was reduced by ≥50% in 58.8% of patients, and 23.5% achieved seizure freedom [66]. Complementing these findings, a larger multicenter study by Gil-Nagel et al. evaluated 82 DEE patients and found a 65% responder rate at 12 months, with no significant efficacy difference between Dravet and non-Dravet patients [67].

Further supporting evidence comes from a Phase II RCT, reported in abstract form, evaluating stiripentol in 16 LGS patients. In this pharmaceutical-sponsored trial, stiripentol was added to existing treatment regimens for two months, at a daily dose between 2000 and 3000 mg depending on patient age. Notably, 9 patients achieved >50% seizure reduction and 5 patients became seizure-free during this period [68].

Seizure type-specific analyses provide additional insight into stiripentol’s utility in LGS. Among non-Dravet patients in one study, ≥50% seizure reduction was achieved in 100% of those with GTCS, 75% with myoclonic seizures, 71.5% with absence seizures, 50% with epileptic spasms, and 33.3% with tonic seizures—patterns highly relevant to LGS management [66].

### 7.2. Safety and Tolerability

Stiripentol demonstrated an acceptable tolerability profile in LGS-relevant populations. In the one study, 47.1% of non-Dravet patients reported AEs, with drowsiness being the most common (17.6%) [66]. Similarly, another study reported AEs in 46% of patients, including somnolence and decreased appetite [67]. In the Phase II RCT, one patient discontinued due to side effects (nausea, vomiting, somnolence) [68]. Other reported AEs included anorexia, nausea, vomiting, constipation, and somnolence [68].

### 7.3. Current Clinical Role

Stiripentol offers several potential advantages in LGS management, particularly through its multimodal mechanism of action and preferential efficacy against certain generalized seizure types. It appears especially beneficial for GTCS, myoclonic seizures, and atypical absence seizures, which are commonly observed in LGS patients [67]. Stiripentol may also have a role in treating status epilepticus, which is frequently encountered in LGS [69].

Of particular clinical importance, stiripentol is a potent inhibitor of CYP2C9 and CYP2C19, leading to significant elevations in N-desmethylclobazam (the active metabolite of clobazam) and, to a lesser extent, clobazam itself [70]. Its ability to augment clobazam efficacy via pharmacokinetic enhancement may provide additional value in LGS patients already treated with clobazam, although this necessitates careful monitoring and dosage adjustments to mitigate side effects. Initiating stiripentol typically requires reduction in clobazam and valproate doses.

Despite these potential benefits, the current lack of robust LGS-specific data places stiripentol lower in the treatment hierarchy. Its use in LGS remains off-label and should be considered cautiously, particularly in patients with prominent GTCS already receiving clobazam or in cases of refractory status epilepticus.

## 8. Conclusions

The newer ASMs—perampanel, brivaracetam, cenobamate, ganaxolone, and stiripentol—offer promising but variable therapeutic options for LGS, each with distinct mechanisms of action and clinical profiles that may help address the complex needs of this heterogeneous population. Perampanel has the most robust evidence base but requires careful monitoring for behavioral AEs. Brivaracetam may serve as a more behaviorally tolerable alternative to levetiracetam, despite mixed efficacy data. Cenobamate demonstrates exceptional promise, including unprecedented seizure freedom rates and potential for polypharmacy reduction, though it is currently approved only for adults and necessitates cautious titration. Stiripentol may be beneficial for GTCS in LGS, while ganaxolone has shown proof-of-concept efficacy in LGS-like phenotypes, particularly in certain genetic epilepsies such as CDKL5 deficiency disorder. Although current evidence is limited by a paucity of RCTs and heterogeneity in outcome measures, these agents represent meaningful therapeutic advances. Their use should be considered within individualized treatment algorithms that account for seizure type, cognitive and behavioral comorbidities, age, and prior treatment responses. Notably, most studies have focused on seizure frequency reduction, with limited evaluation of broader outcomes such as quality of life, cognitive function, and caregiver burden—metrics increasingly recognized as essential in LGS management. Furthermore, most studies do not provide participant-level clinical details, extended long-term follow-up, or genetic information to support precision therapeutics, with possible exceptions for *SCN8A* and *CDKL5*-related epilepsy. Future research should prioritize head-to-head comparative trials, standardized outcome measures, and precision medicine approaches to better inform treatment selection and improve outcomes in this challenging epilepsy syndrome.

## Figures and Tables

**Table 1:** Antiseizure Medications in Lennox–Gastaut Syndrome: Summary of Key Clinical Studies and Outcomes.

Medication (Trial, Duration)	Drop Attack Reduction	≥50% Drop Responder Rate	Total Seizure Reduction	≥50% Total Responder Rate	Long-Term/Extension Outcomes	Global/Clinical Evaluation
Felbamate (Felbamate Study Group; 10 weeks)	34% vs. 9% (*p* = 0.01)	N/A	19% vs. +4% (*p* = 0.002)	N/A	~50% reached ≥50% reduction in total seizures at 12 mo; ~67% had ≥50% reduction in atonic seizures	Significant improvement on global evaluations; improved performance on digit-symbol neuropsychological test
Lamotrigine (Motte et al.; 16 weeks)	34% vs. 9% (*p* = 0.01)	37% vs. 22% (*p* = 0.04)	32% vs. 9% (*p* = 0.002)	33% vs. 16% (*p* = 0.01)	N/A	N/A
Topiramate (Sachdeo et al., Glauser et al.; 11 weeks)	14.8% vs. +5.1% (*p* = 0.041)	28% vs. 14% (*p* = 0.071)	25.8% vs. +5.2% (*p* = 0.015)	33% vs. 8% (*p* = 0.002)	At 6 mo: 55% had ≥50% drop reduction, 15% seizure-free for ≥6 mo	N/A
Rufinamide (Glauser et al., Kluger et al.; 12 weeks)	42.5% vs. 1.4% (*p* < 0.0001)	42.5% vs. 16.7% (*p* = 0.002)	32.7% vs. 11.7% (*p* = 0.0015)	31.1% vs. 10.9% (*p* = 0.0045)	41% achieved ≥50% reduction in total seizures; 48% in tonic-atonic seizures at OLE	No difference in caregiver global evaluation vs. placebo
Rufinamide (Ohtsuka et al. 2014, 2016; 12 weeks)	24.2% vs. 3.3% (*p* = 0.003)	25% vs. 6.7% (*p* = 0.074)	32.9% vs. 3.1% (*p* < 0.001)	N/A	Sustained seizure reduction across 52 weeks; ~44% ≥50% responder at study end	Significant improvement in clinician global impression (*p* = 0.007)
Clobazam (Ng, Conry; 15 weeks)	Dose-dependent: 41–68% vs. 12% (all *p* ≤ 0.012)	43–78% vs. 32% (*p* < 0.016–0.0001)	35–65% vs. 9% (*p* < 0.041–0.0001)	N/A	At 5 years: 85–97% median reduction in drop seizures; 75–85% in total seizures; 47% remained drop-seizure-free	Physician: 46–65% much/very much improved; Caregiver: 42–59% much/very much improved
Cannabidiol (GWPCARE3/4; 14 weeks)	CBD10: 37% vs. 17% (*p* = 0.002); CBD20: 42–44% vs. 17–22% (*p* ≤ 0.0135)	36–44% vs. 14–24% (*p* ≤ 0.0043)	36–41% vs. 14–19% (*p* ≤ 0.009)	N/A	At 48 weeks: median drop seizure reduction 48–60%; total seizure reduction 48–57%	CGI-I: 57–66% improved vs. 34–44% placebo; Subgroup with clobazam showed stronger effect
Fenfluramine (FFA) (Study 1601; 14 weeks)	FFA0.7: 27% vs. 8% (*p* = 0.0165); FFA0.2: 12% (NS)	FFA0.7: 25% vs. 10% (*p* = 0.02); FFA0.2: 28% vs. 10% (*p* = 0.02)	FFA0.7: 26% vs. 8% (*p* = 0.0165); FFA0.2: NS	GTCS: 46–58% reduction vs. 4% placebo	At 15 mo: 50% median reduction in drop seizures; 31% ≥50% responders; tonic seizures −36%	CGI-I: 45% improved vs. 34% placebo; 26% much/very much improved vs. 6% placebo
Valproate (Covanis et al.)	Better outcomes for drop seizures	N/A	≥50% reduction in 55%; complete seizure control in 21%	N/A	Widely used; efficacy strongest in drop attacks	Side effects: weight gain, hepatotoxicity, GI intolerance
Levetiracetam (Kim et al.)	N/A	58% ≥50% responders; 27% seizure-free	N/A	N/A	Minimal cognitive adverse effects	Most common AE: hyperactivity (13%)
Zonisamide (You et al.)	N/A	60% with >50% seizure reduction; 4.8% seizure free	N/A	N/A	Some patients achieved complete seizure freedom (5%)	Adverse events: somnolence, anorexia (transient)
Lacosamide (Grosso et al., Andrade-Machado et al.)	Variable; higher for focal tonic (~75%) than atonic (~20%)	33% responders (children); lower in adults	Overall seizure reduction ~29%	N/A	Mixed results, occasional worsening of tonic/atonic seizures	Adverse events in ~44%

N/A: Not available.

**Table 2:** PICO Framework for Evaluating Newer Anti-Seizure Medications in Lennox–Gastaut Syndrome.

PICO Element	Description
Population (P)	Patients diagnosed with Lennox–Gastaut syndrome (LGS) or LGS-like epileptic encephalopathies
Intervention (I)	Treatment with newer anti-seizure medications: perampanel, brivaracetam, cenobamate, stiripentol, or ganaxolone
Comparison (C)	Placebo, standard care, or pre-treatment baseline (for observational studies)
Outcomes (O)	Primary: Seizure frequency reduction (≥50% responder rate) Secondary: Seizure freedom, seizure-type specific responses, adverse events, retention rates, quality of life measures
Research Question	In patients with Lennox–Gastaut syndrome, what is the efficacy and safety of newer anti-seizure medications (perampanel, brivaracetam, cenobamate, stiripentol, and ganaxolone) compared to standard treatment approaches?

**Table 3:** Overview of Perampanel, Brivaracetam, Cenobamate, Stiripentol, and Ganaxolone.

Medication	Mechanism/Clinical Action	Dose	Forms	Pharmacokinetics (T½/Metabolism)	Drug Interactions	FDA Indications	Common Side Effects	Age Approved	DEA Schedule	Renal and Hepatic Dosing Considerations
Perampanel	Non-competitive AMPA receptor antagonist; reduces excitatory glutamate transmission	Start 2 mg QHS; titrate to 8–12 mg/day	Oral tablet, oral suspension	T½ ~105 h; CYP3A4 metabolism; ~95% protein bound	↓ levels with CYP3A4 inducers (e.g., carbamazepine); additive sedation with CNS depressants	Adjunctive or monotherapy for focal seizures (≥4 years); adjunctive for PGTC seizures (≥12 years)	Dizziness, irritability, gait disturbance, aggression	≥4 years	Schedule III	Renal: CrCl ≥ 30 mL/min, no adjustment; monitor/titrate slowly if 30–49 mL/min; CrCl < 30 mL/min or dialysis not recommended. Hepatic: Mild (Child-Pugh A): start 2 mg/day, titrate by 2 mg every ≥2 weeks, max 6 mg/day; Moderate (B): max 4 mg/day; Severe (C): not recommended.
Brivaracetam	High-affinity SV2A ligand; modulates neurotransmitter release	50–100 mg BID; Max: 200 mg/day	Oral tablet, oral solution, IV	T½ ~9 h; metabolized by hydrolysis and CYP2C19; ~20% protein bound	↑ levels with CYP2C19 inhibitors; minimal interaction profile	Adjunctive treatment for focal seizures (≥1 month)	Somnolence, fatigue, dizziness, nausea	≥1 month	Schedule V	Renal: Mild to severe impairment, no adjustment; dialysis not recommended. Hepatic: Mild to severe (A–C): initial 25 mg BID, up to 75 mg BID. Older adults: consider starting at low end of range.
Cenobamate	Enhances fast Na^+^ channel inactivation; positive allosteric modulator of GABA-A	Start 12.5 mg/day; titrate to 200 mg/day; Max 400 mg/day	Oral tablet	T½ ~50–60 h; metabolized by CYP2E1, 2A6, 2B6, 2C19, 3A4	Inhibits CYP2C19 (↑ phenytoin, clobazam); induces CYP3A4 (↓ oral contraceptive efficacy)	Focal seizures in adults	Somnolence, dizziness, fatigue, DRESS (boxed warning)	Adults (≥18 years)	Schedule V	Renal and Hepatic: No formal dosing guidance provided; caution advised in moderate-severe impairment; monitor response and tolerability.
Stiripentol	GABA-A receptor modulator; inhibits GABA reuptake and CYP enzymes	50 mg/kg/day in 2–3 divided doses; Max ~3000 mg/day	Oral capsule, oral powder for suspension	T½ ~4.5–13 h; nonlinear kinetics; strong CYP inhibitor (1A2, 2C19, 3A4)	↑ clobazam, valproate levels; risk of sedation; CYP inhibition	Adjunctive treatment of Dravet syndrome with clobazam (≥6 months)	Somnolence, decreased appetite, ataxia	≥2 years	Not scheduled	Renal: No formal guidance; use with caution in mild impairment, avoid moderate-severe. Hepatic: No formal guidance; use with caution in mild impairment, avoid moderate-severe; titrate based on seizure control and tolerability.
Ganaxolone	Neurosteroid; positive allosteric GABA-A modulator	~63 mg/kg/day in 3 divided doses; Max ~1800 mg/day	Oral suspension	T½ ~30–60 h; CYP3A4 metabolism; ~99% protein bound	CYP3A4 inhibitors ↑ levels; inducers ↓ levels	CDKL5 deficiency disorder (≥2 years)	Somnolence, respiratory depression, sedation	≥2 years	Schedule V	Renal: No adjustment required; minimal renal elimination. Hepatic: Mild-moderate (A/B): no adjustment; Severe (C >28 kg): titrate weekly from 50 mg TID → 200 mg TID by week 4.

↓: decrease; ↑: increase.

## Data Availability

No new data were created or analyzed in this study. Data sharing is not applicable to this article.

## Supplementary Materials

The following supporting information can be downloaded at: https://www.mdpi.com/article/10.3390/jcm14176302/s1, Figure S1. PRISMA flow diagram showing the systematic literature search and study selection process.

## Abbreviations

AMPA	α-amino-3-hydroxy-5-methyl-4-isoxazolepropionic acid
BID	Twice daily
CNS	Central nervous system
CYP	Cytochrome P450 enzyme
DRESS	Drug Reaction with Eosinophilia and Systemic Symptoms
GABA	Gamma-aminobutyric acid
IV	Intravenous
PK	Pharmacokinetics
PGTC	Primary Generalized Tonic–Clonic
QHS	Every night at bedtime
SV2A	Synaptic vesicle protein 2A
T½	Elimination half-life
