# Supplementary material for: Perampanel, Brivaracetam, Cenobamate, Stiripentol, and Ganaxolone in Lennox-Gastaut Syndrome: A Comprehensive Narrative Review
Source: J Clin Med. 2025 Sep 6;14(17):6302. doi: 10.3390/jcm14176302 (PMC12429675; doi:10.3390/jcm14176302)
Supplement: Supplementary file 1 [file jcm-14-06302-s001.zip › jcm-3820600-supplementary.pdf]

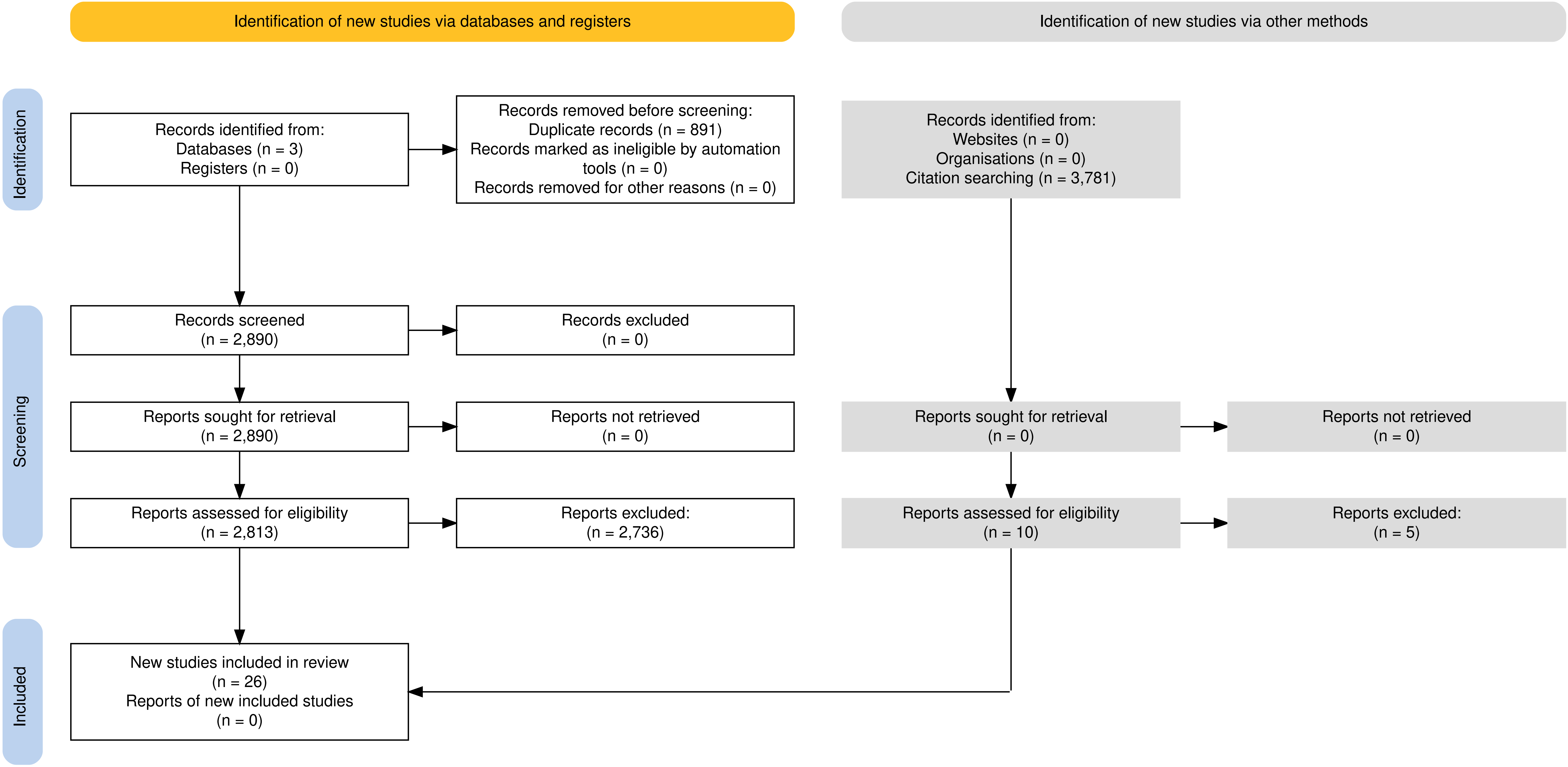
Identification of new studies via databases and registers
Identification of new studies via other methods
Identification
Screening
Included
Records identified from:
Databases (n = 3)
Registers (n = 0)
Records removed before screening:
Duplicate records (n = 891)
Records marked as ineligible by automation tools (n = 0)
Records removed for other reasons (n = 0)
Records identified from:
Websites (n = 0)
Organisations (n = 0)
Citation searching (n = 3,781)
Records screened
(n = 2,890)
Records excluded
(n = 0)
Reports sought for retrieval
(n = 2,890)
Reports not retrieved
(n = 0)
Reports sought for retrieval
(n = 0)
Reports not retrieved
(n = 0)
Reports assessed for eligibility
(n = 2,813)
Reports excluded:
(n = 2,736)
Reports assessed for eligibility
(n = 10)
Reports excluded:
(n = 5)
New studies included in review
(n = 26)
Reports of new included studies
(n = 0)
